# *TP53* genetic polymorphisms, interactions with lifestyle factors and lung cancer risk: a case control study in a Chinese population

**DOI:** 10.1186/1471-2407-13-607

**Published:** 2013-12-27

**Authors:** Yanli Li, Shen-Chih Chang, Rungui Niu, Li Liu, Christina R Crabtree-Ide, Baoxing Zhao, Jianping Shi, Xiaoyou Han, Jiawei Li, Jia Su, Lin Cai, Shunzhang Yu, Zuo-Feng Zhang, Lina Mu

**Affiliations:** 1 Department of Social and Preventive Medicine, School of Public Health and Health Professions, The State University of New York (SUNY) at Buffalo, 273A Farber Hall, Buffalo, New York 14214-8001, USA; 2 Department of Epidemiology, Fielding School of Public Health, University of California, Los Angeles (UCLA), Los Angeles, CA, USA; 3 Shanxi Tumor Hospital, Taiyuan, Shanxi, Province, China; 4 Taiyuan City Center for Disease Control and Prevention (CDC), Taiyuan, Shanxi, Province, China; 5 School of Public Health, Fudan University, Shanghai, China; 6 Department of Epidemiology, School of Public Health, Fujian Medical University, Fuzhou, Fijian, China

**Keywords:** Lung cancer, TP53, Single-nucleotide polymorphism, Chinese population

## Abstract

**Background:**

A pathway-based genotyping analysis suggested rs2078486 was a novel *TP53* SNP, but very few studies replicate this association. *TP53* rs1042522 is the most commonly studied SNP, but very few studies examined its potential interaction with environmental factors in relation to lung cancer risk. This study aims to examine associations between two *TP53* single-nucleotide polymorphisms (SNPs) (rs2078486, rs1042522), their potential interaction with environmental factors and risk of lung cancer.

**Methods:**

A case–control study was conducted in Taiyuan, China. Unconditional logistic regression was used to estimate odds ratios (ORs) and 95% confidence intervals (95% CIs). Multiplicative and additive interactions between *TP53* SNPs and lifestyle factors were evaluated.

**Results:**

Variant *TP53* rs2078486 SNP was significantly associated with elevated lung cancer risk among smokers (OR: 1.70, 95% CI: 1.08 - 2.67) and individuals with high indoor air pollution exposure (OR: 1.51, 95% CI: 1.00-2.30). Significant or borderline significant multiplicative and additive interactions were found between *TP53* rs2078486 polymorphism with smoking and indoor air pollution exposure. The variant genotype of *TP53* SNP rs1042522 significantly increased lung cancer risk in the total population (OR: 1.57, 95% CI: 1.11-2.21), but there was no evidence of heterogeneity among individuals with different lifestyle factors.

**Conclusions:**

This study confirmed that *TP53* rs2078486 SNP is potentially a novel *TP53* SNP that may affect lung cancer risk. Our study also suggested potential synergetic effects of *TP53* rs2078486 SNP with smoking and indoor air pollution exposure on lung cancer risk.

## Background

Lung cancer is one of the most common cancers and is a leading cause of cancer death in China. It was estimated that by year 2025, more than one million Chinese will be diagnosed with lung cancer per year [1]. Lung cancer mortality increased 465% during the past 30 years and now is the leading cancer death cause in China [2]. Smoking is regarded as the most important risk factor for lung cancer, and indoor air pollution from cooking and heating is another potential risk factor in Chinese population [3]. However, approximately one in ten lifetime smokers develop lung cancer, which implies a possible role for genetic susceptibility in the development of lung cancer [4].

The *TP53* tumor suppressor gene plays a critical role in modulating transcription of genes that govern the major defenses against tumor growth, including cell cycle arrest, apoptosis, maintenance of genetic integrity, inhibition of angiogenesis and cellular senescence [5]. The *TP53* gene harbors high-frequency, functional single-nucleotide polymorphisms (SNPs) which may alter P53 protein function [6]. Several functional *TP53* SNPs have been reported to be associated with risk of developing different human cancers, including lung cancer [7-9].

*TP53* rs2078486 SNP was recently identified to be associated with lung cancer risk in lifetime never smokers in a pathway-based genotyping study which evaluated a comprehensive panel of 11,737 SNPs in inflammatory-pathway genes [10]. One case–control study conducted among 611 lung cancer cases and 1040 controls in Los Angeles found elevated lung cancer risk associated with the variant genotype of *TP53* rs2078486 SNP (doctoral dissertation from Yi Ren Wang) [11]. However this association was not confirmed by another pooled genome-wide association study [12]. In addition to lung cancer, *TP53* rs2078486 SNP has been also linked with risk of ovarian cancer [13] and schizophrenia [14]. To our knowledge, no case–control study has been conducted in the Asian population to replicate the association of *TP53* rs2078486 SNP with lung cancer.

The most studied *TP53* SNP rs1042522 is characterized by substitution of Arginine (Arg) by Proline (Pro) at codon 72 (G12139C, Arg72Pro) and may noticeably affect P53 function [15]. However, very few studies examined if there are interactions between Arg72Pro polymorphism and smoking or other lifestyle factors on lung cancer risk.

A case–control study was conducted to examine the associations of *TP53* rs2078486 and rs1042522 SNPs with lung cancer risk in a Chinese population and further explore their interactions with some demographic and lifestyle factors.

## Methods

### Study participants

A case–control study was conducted between 2005 and 2007 in Taiyuan city, the capital of Shanxi province, China. The original study population has been described in detail previously [16]. Prior to the initiation of the recruitment, IRB approvals were obtained from Fudan University (IRB#04-10-0022) and UCLA (IRB#11-003153), respectively. Lung cancer cases were enrolled from the Shanxi tumor hospital, which admitted about 70% of the cancer patients from the city. Eligible cases were newly diagnosed lung cancer cases, 20 years of age or older, lived in Taiyuan city for 10 years or more, in stable medical condition and willing to participate. Controls were randomly selected from 13 communities in Taiyuan city. Eligible controls were 20 years of age or older, must have lived in Taiyuan city for 10 years or more, and had no history of cancer or any other serious chronic diseases. A total of 399 lung cancer patients and 466 healthy controls were recruited to participate in this study. Response rates were 89% for eligible cases and 85% for eligible controls. Written informed consent was obtained from all study participants.

### Data collection

All cases and controls were interviewed by professional staff to collect information on demographic factors, dietary and cooking habits, active and passive smoking history, alcohol drinking habits, tea drinking habits, residence and housing history, occupational history and related exposure, physical activities and disease history.

### Blood sample collection and laboratory analysis of gene polymorphisms

Blood samples were collected from 97.9% of cases and 98.9% of controls. Serum and blood clot were immediately separated and all samples were stored in freezer at −80°C. Genomic DNA was extracted using a modified phenol-chloroform protocol. Genotyping was performed in the Molecular Epidemiology Laboratory at Department of Epidemiology, School of Public Health at UCLA. *TP53* SNP genotyping was performed using Sequenom platform (Sequenom, Inc., San Diego, CA). Polymerase chain reaction (PCR) and extension primers were designed using MassARRAY Assay Design 3.1 software (Sequenom, Inc., San Diego, CA). Genotyping procedures were performed according to the manufacturer’s iPLEX Application Guide (Sequenom Inc. SanDiego,CA). For quality control, we included two negative controls (H_2_O) in each 96-well plate. Around 4.5% of samples were selected for duplication and the concordance is 99.5%. We found no obvious deviations from Hardy-Weinberg equilibrium for both SNPs (rs2078486: χ^2^ = 0.19, P = 0.6629; rs1042522: χ^2^ = 4.24, P = 0.0395) among control subjects. We did not find strong linkage disequilibrium between the two SNPs (D’ < 0.5) in the current study and this is consistent with previous studies [17,18].

### Definition of indoor air pollution index

An indoor air pollution index was created to integrate the impacts from different types of cooking and heating fuels, use of ventilator in kitchen, windows opening behaviors and secondhand smoke exposure at home on indoor air pollution levels. For each component of this index, a score of ‘0′ or ‘1′ represented low or high indoor air pollution, respectively. A summarized score lower than 2 was defined as low indoor air pollution exposure and higher or equal to 2 was defined as high indoor air pollution exposure [16].

### Statistical analysis

Odds ratios (ORs) and 95% confidence intervals (95% CIs) were estimated using unconditional logistic regression models to evaluate the independent effects of the two *TP53* SNPs. We presented each of the associations in additive, dominant and recessive models, respectively. Potential confounding factors adjusted in the multivariate models included age, education level, annual personal income 10 years ago, pack-years of smoking, alcohol drinking and tea drinking status. Stratified analyses were conducted among subgroups with different age, gender, smoking status, alcohol and tea drinking status, indoor air pollution exposure and histo-pathological types of lung cancer. Multiplicative interactions of *TP53* SNPs with some lifestyle factors were assessed using ORs for interactions by including their product terms in the logistic regression models. Additive interactions were assessed using relative excess risk due to interaction (RERI), as described previously [19]. All statistical analyses were performed using SAS software (version 9.3). Associations were considered statistically significant if the p-value < 0.05 in the two-sided test.

## Results

Basic characteristics of lung cancer cases and controls are presented in Table 1. No statistically significant differences in age and gender were found between cases and controls. Controls tended to have higher education levels, average annual income and body mass index than cases (p < 0.0001). Lung cancer cases were more likely to be smokers and had higher pack-years of smoking, but were less likely to be current tea drinkers (p < 0.0001) (Table 1).

**Table 1:** Basic characteristics of cases and controls in Taiyuan lung cancer study

**Variable**	**Cases**	**Controls**	**P value**
	**N (%)**	**N (%)**	
**Age**			
≤ 45 years	59 (14.8)	83 (17.8)	0.0725
45 – 55 yrs	96 (24.1)	139 (29.8)	
55 – 65 yrs	111 (27.8)	116 (25.0)	
> 65 yrs	133 (33.3)	128 (27.4)	
**Gender**			
Male	202 (50.6)	234 (50.2)	0.9038
Female	197 (49.4)	232 (49.8)	
**Education**			
Illiterate	43 (10.8)	23 (4.9)	**<.0001**
Primary school	106 (26.6)	81 (17.4)	
Junior middle school	124 (31.1)	175 (37.5)	
Senior middle school	68 (17.0)	120 (25.8)	
College or higher	58 (14.5)	67 (14.4)	
**Pack years of smoking**			
Non smokers	179 (44.9)	285 (61.2)	**<.0001**
< 20 pyrs	39 (9.8)	62 (13.3)	
20 – 40 pyrs	64 (16.0)	72 (15.5)	
≥ 40 pyrs	117 (29.3)	47 (10.1)	
**Average income 10 years ago**			
< 1,000 yuan	104 (26.1)	106 (22.7)	**<.0001**
1,000 – 2,500 yuan	236 (59.1)	197 (42.3)	
≥ 2,500 yuan	59 (14.8)	163 (35.0)	
**BMI (kg/m**^**2**^**)**			
< 18.5	22 (5.8)	9 (2.0)	**<.0001**
18.5 – 24.9	250 (66.3)	259 (56.3)	
25 – 29.9	90 (23.9)	162 (35.2)	
≥ 30	15 (4.0)	30 (6.5)	
**Alcohol drinking**			
No	298 (74.7)	345 (74.0)	0.8267
Yes	101 (25.3)	121 (26.0)	
**Tea drinking**			
No	242 (60.6)	263 (56.5)	**<.0001**
Previous drinkers	47 (11.8)	15 (3.2)	
Current drinkers	110 (27.6)	188 (40.3)	
**Total**	399	466	

Adjusted age, gender, education, pack years of smoking, alcohol drinking, tea drinking and average income 10 years ago.

Significant differences were highlighted in bold.

Table 2 presents the independent associations between the two *TP53* SNPs and lung cancer risk in the total study population. No significant associations with lung cancer risk were found for *TP53* rs2078486 SNP, despite a tendency towards an elevated lung cancer risk associated with the variant genotype. A significantly increased lung cancer risk was observed among individuals with the homozygous variant genotype (CC) of *TP53* SNP rs1042522 (adjusted OR: 1.63, 95% CI: 1.10 - 2.41), compared with the homozygous wild type (GG). Adjusted ORs for rs1042522 were also statistically significant in the recessive model (adjusted OR: 1.57, 95% CI: 1.11- 2.21), but not in the dominant model. C allele of *TP53* SNP rs1042522 was significantly associated with increased risk of developing lung cancer (adjusted OR: 1.26, 95% CI: 1.04 - 1.53) (Table 2).

**Table 2:** **Associations of *TP53 *** **SNPs with lung cancer risk in Taiyuan lung cancer study in total study population**

**SNP**	**Genotype**	**Cases**		**Controls**		**Crude OR (95% CI)**	Adjusted OR* (95% CI)
		**N**	**%**	**N**	**%**		
***TP53 *** **rs2078486**		355		448			
	CC	186	52.4	250	55.8	1	1
	TC	136	38.3	167	37.3	1.10 (0.82, 1.47)	1.20 (0.88, 1.64)
	TT	33	9.3	31	6.9	1.43 (0.85, 2.42)	1.30 (0.75, 2.25)
Dominant model	Any T vs. CC	169	47.6	198	44.2	1.15 (0.87, 1.52)	1.22 (0.91, 1.63)
Recessive model	TT vs. Any C	33	9.3	31	6.9	1.38 (0.83, 2.30)	1.20 (0.70, 2.06)
Allele OR	T vs. C					1.15 (0.93, 1.43)	1.17 (0.93, 1.46)
***TP53 *** **rs1042522**		363		446			
	GG	118	32.5	161	36.1	1	1
	CG	146	40.2	196	43.9	1.02 (0.74, 1.40)	1.07 (0.77, 1.50)
	CC	99	27.3	89	20.0	**1.52 (1.05, 2.20)**	**1.63 (1.10, 2.41)**
Dominant model	Any C vs. GG	245	67.5	285	63.9	1.17 (0.88, 1.57)	1.24 (0.92, 1.69)
Recessive model	CC vs. Any G	99	27.3	89	20.0	**1.50 (1.08, 2.09)**	**1.57 (1.11, 2.21)**
Allele OR	C vs. G					**1.21 (1.01, 1.46)**	**1.26 (1.04, 1.53)**

*Adjusted for age, gender, education, pack years of smoking, alcohol drinking, tea drinking and average income 10 years ago.

Significant results were highlighted in bold.

**Figure 1:**
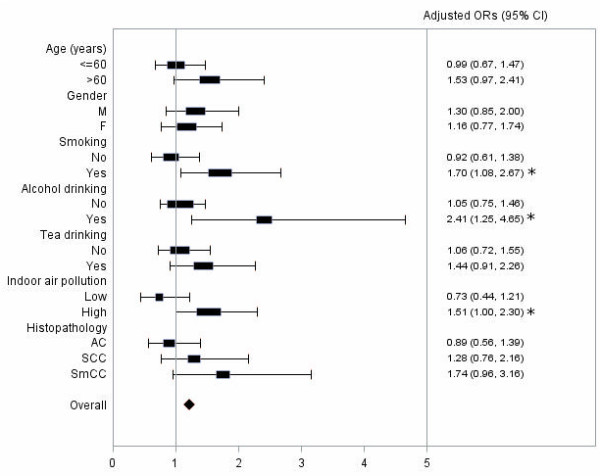
**Associations of *TP53 *rs2078486 with lung cancer among different subgroups with different age, gender, smoking status, alcohol and tea drinking status, indoor air pollution exposure and histo-pathological types of lung cancer.** Adjusted age, gender, education, pack years of smoking, alcohol drinking, tea drinking and average income 10 years ago. AC: adenocarcinoma, SCC: squamous cell carcinoma; SmCC: small cell carcinoma. Asterisks indicate significant ORs.

Results from the stratified analyses are presented in Figure 1. Presence of one or both copies of minor allele (TC or CC) of *TP53* rs2078486 SNP was significantly or borderline significantly associated with elevated lung cancer risk among older individuals (adjusted OR: 1.53, 95% CI: 0.97 - 2.41), smokers (adjusted OR: 1.70, 95% CI: 1.08 - 2.67), alcohol drinkers (adjusted OR: 2.41, 95% CI: 1.25 - 4.65) and individuals with high indoor air pollution exposure (adjusted OR: 1.51, 95% CI: 1.00-2.30) (Figure 1). Significant multiplicative and additive interactions were found between the indoor air pollution index and *TP53* rs2078486 polymorphism (adjusted OR for interaction: 1.89, 95% CI: 1.00-3.56, adjusted REPI: 0.90, 95% CI: 0.11-1.70). There was also some suggestive evidence of multiplicative interaction between smoking and *TP53* rs2078486 polymorphism (adjusted OR for interaction: 1.80, 95% CI: 0.99-3.30) and additive interaction (adjusted RERI: 2.49, 95% CI: -0.03, 5.01) (Table 3).

**Table 3:** **Interaction between *TP53 *** **SNPs and lifestyle factors in Taiyuan lung cancer study**

		**Cases**	**Controls**	**Crude OR (95% CI)**	Adjusted OR* (95% CI)
**Smoking**	**rs2078486**				
No	CC	95	147	1	1
No	TC or TT	70	127	0.85 (0.58, 1.26)	0.93 (0.62, 1.39)
Yes	CC	91	103	1.37 (0.93, 2.00)	**3.58 (2.02, 6.36)**
Yes	TC or TT	99	71	**2.16 (1.45, 3.22)**	**6.00 (3.33, 10.81)**
OR for interaction				**1.85 (1.05, 3.27)**	1.80 (0.99, 3.30)
RERI				**0.94 (0.14, 1.74)**	2.49 (−0.03, 5.01)
**Alcohol drinking**	**rs2078486**				
No	CC	147	184	1	1
No	TC or TT	124	148	1.05 (0.76, 1.45)	1.06 (0.76, 1.48)
Yes	CC	39	66	0.74 (0.47, 1.16)	0.71 (0.42, 1.21)
Yes	TC or TT	45	50	1.13 (0.71, 1.78)	1.35 (0.79, 2.30)
OR for interaction				1.45 (0.76, 2.78)	1.79 (0.90, 3.58)
RERI				0.34 (−0.27, 0.95)	0.58 (−0.15, 1.30)
**Tea drinking**	**rs2078486**				
Yes	CC	65	107	1	1
Yes	TC or TT	75	91	1.36 (0.88, 2.09)	1.44 (0.91, 2.28)
No	CC	121	143	1.39 (0.94, 2.06)	1.43 (0.92, 2.22)
No	TC or TT	94	107	1.45 (0.96, 2.19)	1.51 (0.94, 2.41)
OR for interaction				0.77 (0.43, 1.35)	0.73 (0.40, 1.32)
RERI				−0.30 (−1.08, 0.48)	−0.37 (−1.21, 0.48)
**Indoor air pollution**	**rs2078486**				
Low	CC	63	124	1	1
Low	TC or TT	41	105	0.77 (0.48, 1.23)	0.78 (0.48, 1.28)
High	CC	107	122	**1.73 (1.16, 2.57)**	**1.42 (0.92, 2.20)**
High	TC or TT	104	89	**2.30 (1.52, 3.48)**	**2.11 (1.35, 3.29)**
OR for interaction				1.73 (0.94, 3.18)	**1.89 (1.00, 3.56)**
RERI				0.81 (−0.03, 1.65)	**0.90 (0.11, 1.70)**
**Smoking**	**rs1042522**				
No	GG or CG	120	216	1	1
No	CC	48	58	1.49 (0.96, 2.32)	**1.59 (1.00, 2.53)**
Yes	GG or CG	144	141	**1.84 (1.33, 2.54)**	**4.73 (2.78, 8.04)**
Yes	CC	51	31	**2.96 (1.80, 4.88)**	**8.00 (4.08, 15.71)**
OR for interaction				1.08 (0.55, 2.11)	1.07 (0.52, 2.18)
RERI				0.63 (−0.87, 2.14)	2.69 (−1.64, 7.02)
**Alcohol drinking**	**rs1042522**				
No	GG or CG	200	263	1	1
No	CC	74	69	1.41 (0.97, 2.05)	1.45 (0.98, 2.14)
Yes	GG or CG	64	94	0.90 (0.62, 1.29)	0.90 (0.57, 1.41)
Yes	CC	25	20	1.64 (0.89, 3.04)	1.86 (0.92, 3.76)
OR for interaction				1.30 (0.61, 2.80)	1.44 (0.63, 3.27)
RERI				0.34 (−0.78, 1.45)	0.52 (−0.82, 1.86)
**Tea drinking**	**rs1042522**				
Yes	GG or CG	100	159	1	1
Yes	CC	41	35	**1.86 (1.11, 3.12)**	**2.09 (1.21, 3.61)**
No	GG or CG	164	198	1.32 (0.95, 1.82)	1.39 (0.95, 2.04)
No	CC	58	54	**1.71 (1.09, 2.67)**	**1.81 (1.10, 2.97)**
OR for interaction				0.70 (0.36, 1.36)	0.62 (0.31, 1.25)
RERI				−0.47 (−1.62, 0.68)	−0.67 (−1.99, 0.65)
**Indoor air pollution**	**rs1042522**				
Low	GG or CG	73	188	1	1
Low	CC	33	41	**2.07 (1.22, 3.53)**	**1.99 (1.14, 3.48)**
High	GG or CG	159	163	**2.51 (1.78, 3.56)**	**2.04 (1.40, 2.99)**
High	CC	56	46	**3.14 (1.95, 5.04)**	**2.76 (1.66, 4.58)**
OR for interaction				0.60 (0.30, 1.21)	0.68 (0.33, 1.41)
RERI				−0.45 (−2.14, 1.24)	−0.28 (−1.88, 1.33)

*Adjusted for age, gender, education, pack years of smoking, alcohol drinking, tea drinking and average income 10 years ago.

Significant results were highlighted in bold.

**Figure 2:**
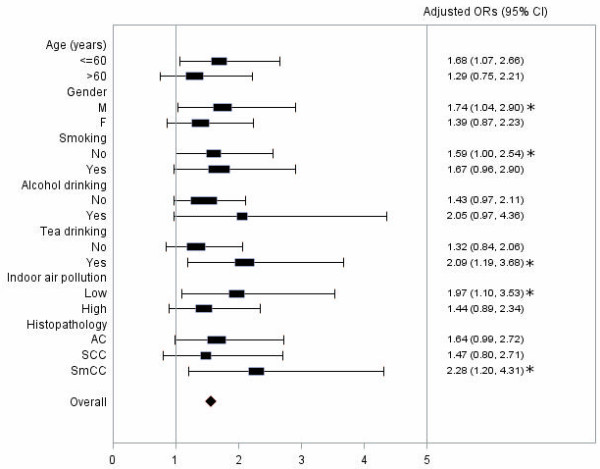
**Associations of *TP53 *rs1042522 with lung cancer among different subgroups with different age, gender, smoking status, alcohol and tea drinking status, indoor air pollution exposure and histo-pathological types of lung cancer.** Asterisks indicate significant ORs. AC: adenocarcinoma, SCC: squamous cell carcinoma; SmCC: small cell carcinoma. Adjusted age, gender, education, pack years of smoking, alcohol drinking, tea drinking and average income 10 years ago.

Elevated risk of lung cancer associated with homozygous variant genotype (CC) of *TP53* SNP rs1042522 were observed in each subgroup. No obvious difference was observed between smokers and nonsmokers (Figure 2). The variant genotype of *TP53* SNP rs1042522 tended to confer stronger deleterious effect for younger individuals, males, alcohol and tea drinkers, however neither multiplicative nor additive interactions were observed between *TP53* SNP rs1042522 and any lifestyle factors on lung cancer risk (Table 3).

## Discussion

This case–control study confirmed elevated lung cancer risk associated with the variant allele (C) of *TP53* SNP rs1042522, and this study is among the first to report a tendency of increased lung cancer risk associated with variant genotype of *TP53* SNP rs2078486 in an Asian population. Moreover, we found synergetic effects of smoking and indoor air pollution exposure with TP53 SNP rs2078486 on lung cancer risk.

Overwhelming evidence suggested that the *TP53* tumor suppressor gene is a central regulatory node of multiple cellular response pathways to endogenous or exogenous stresses [20]. P53 protein has demonstrated the capacity to regulate activity of key effectors of cellular processes, such as DNA repair, cell cycle arrest, senescence, and apoptosis [21,22]. Functional inactivation of P53 pathways is thought to affect P53 signaling and further alter cancer risk [20,23].

*TP53* rs2078486 SNP might be a novel *TP53* SNP affecting risk of developing lung cancer. One pathway-based genotyping study conducted among nonsmokers found statistically significant association between *TP53* rs2078486 SNP and lung cancer [10]. In the present study, we found some suggestive evidence of elevated lung cancer risk associated with TC or CC genotypes of *TP53* rs2078486 SNP (adjusted OR: 1.22, 95% CI: 0.91 -1.63) in the overall study participants. Our result was in similar direction with one population-based case–control study conducted in Los Angeles (adjusted OR: 1.61, 95% CI: 1.18 – 2.20) [11]. The less significant association observed in our study might relate to the relatively smaller sample size. Some other case–control studies also suggested that variant genotype of *TP53* rs2078486 SNP was significantly associated with increased risks of ovarian cancer and schizophrenia [13,14,24]. Therefore, this suggests that there might be a functional difference among different genotypes of *TP53* rs2078486 SNP, which may affect the risk of developing various types of cancers and other human diseases.

*TP53* rs2078486 is located in intron 1 and thus is not likely to be a direct disease-causing polymorphism. However previous studies suggest that *TP53* rs2078486 is in a large linkage disequilibrium block extending from upstream of exon 1 to the first half of intron 1 [17]. Therefore it is possible that *TP53* rs2078486 might be in linkage disequilibrium with some functional polymorphisms, which in turn alter susceptibility to human diseases. Some other intronic variations in *TP53* were reported to affect disease risk previously and the most widely studied one is *TP53* intron 3 duplication polymorphism (rs17878362) [5,25]. The underlying mechanism by which *TP53* rs2078486 modulates cancer risk is not fully understood and warrants further investigations. However prior studies provided some initial evidence that *TP53* rs2078486 is in perfect linkage disequilibrium with *TP53* rs2287498, which is predicted to affect function at a splice site and *TP53* rs2078486 is also in weak linkage disequilibrium with *TP53* rs12951953, which might affect a transcription factor binding site [13].

Moreover, we found carrying the variant alleles of *TP53* rs2078486 SNP was significantly associated with elevated lung cancer risk in smokers (adjusted OR: 1.70, 95% CI: 1.08 - 2.67) and individuals with high indoor air pollution exposure (adjusted OR: 1.51, 95% CI: 1.00-2.30). Cigarette smoking and air pollution have been linked with high frequency of *TP53* mutations [26-29]. The positive interactions observed between *TP53* SNP rs2078486 with smoking and indoor air pollution exposure in our study might suggest that individuals carrying the variant genotype of *TP53* rs2078486 may have compromised P53 function and respond poorly to the adverse effects of smoking and air pollution, thus have an elevated risk of developing lung cancer. In addition, the elevated risk associated with high-risk genotypes *TP53* rs2078486 SNP was more evident for the small cell carcinoma, which has been more strongly linked to cigarette smoking than the other histo-pathological types of lung cancer.

Elevated lung cancer risk associated with the variant C allele of *TP53* SNP rs1042522 observed in this study was consistent with previous studies conducted among the Asian populations (summarized OR under recessive genetic model: 1.37, 95% CI: 1.20 – 1.57; homozygote comparison CC vs. GG: 1.34, 95% CI: 1.16 – 1.56) [8]. In the present study, we did not find heterogeneity of lung cancer risks associated with *TP53* SNP rs1042522 in smokers versus non-smokers, which was also consistent with a previous meta-analysis [8]. Very few prior studies have examined if demographic or other lifestyle factors might modify the association between *TP53* SNP rs1042522 and lung cancer. In this study, we did not find statistically significant interactions between lifestyle factors and *TP53* SNP rs1042522 on lung cancer risk.

One major limitation of the present study is that the relatively small sample size, especially in the stratified analyses, limited our ability to detect moderate interactions. Large-scale epidemiological studies are needed in the future to confirm our findings. Second, after conducting Bonferroni correction for multiple comparisons, no significant interactions between lifestyle factors and *TP53* rs2078486 SNP remained; therefore we cannot exclude the possibility of spurious associations due to multiple comparisons. Lastly, recall bias is likely for established or probable risk factors of lung cancer, such as smoking and air pollution, in a case–control study. However the association between smoking and lung cancer observed in the current study is similar to the previous studies conducted in an Asian population [30]. To minimize the possible recall bias on indoor air pollution exposure, we collected information on several relevant variables, such as cooking, heating and window opening behaviors.

## Conclusions

In conclusion, this case–control study provided preliminary evidence that *TP53* rs2078486 SNP is a novel *TP53* SNP that may affect lung cancer risk, especially among smokers and individuals with high indoor air pollution exposure. There is some further evidence of significant interactions between *TP53* rs2078486 SNP and smoking and indoor air pollution exposure on lung cancer risk. Further studies with larger sample size and in different study populations are warranted to confirm our findings.

## Abbreviations

SNPs: Single-nucleotide polymorphisms; ORs: Odds ratios; 95% CIs: 95% confidence intervals; RERI: Relative excess risk due to interaction.

## Competing interests

The authors declare that they have no competing interests.

## Authors’ contributions

YL performed the statistical analysis and drafted the manuscript. SC carried out the genetic polymorphism tests and helped to draft the manuscript. RN, LL, BZ, JS and XH have made substantial contributions to fieldwork and data collection. CC helped to draft the manuscript. JL, JS and LC participated in the design and coordination of the study. SY and ZZ participated in the design and fieldwork of the study. LM oversaw the study design, results interpretation and manuscript drafting. All authors read and approved the final manuscript.

## Pre-publication history

The pre-publication history for this paper can be accessed here:

http://www.biomedcentral.com/1471-2407/13/607/prepub
